# Development of a Kalman Filter in the Gauss-Helmert Model for Reliability Analysis in Orientation Determination with Smartphone Sensors

**DOI:** 10.3390/s18020414

**Published:** 2018-01-31

**Authors:** Andreas Ettlinger, Hans Neuner, Thomas Burgess

**Affiliations:** 1 Department of Geodesy and Geoinformation, TU Wien, 1040 Wien, Austria; hans.neuner@geo.tuwien.ac.at; 2 indoo.rs GmbH, 1150 Wien, Austria; thomas@indoo.rs

**Keywords:** Kalman filter, Gauss-Helmert model, reliability, partial redundancy, orientation determination, indoor navigation

## Abstract

The topic of indoor positioning and indoor navigation by using observations from smartphone sensors is very challenging as the determined trajectories can be subject to significant deviations compared to the route travelled in reality. Especially the calculation of the direction of movement is the critical part of pedestrian positioning approaches such as Pedestrian Dead Reckoning (“PDR”). Due to distinct systematic effects in filtered trajectories, it can be assumed that there are systematic deviations present in the observations from smartphone sensors. This article has two aims: one is to enable the estimation of partial redundancies for each observation as well as for observation groups. Partial redundancies are a measure for the reliability indicating how well systematic deviations can be detected in single observations used in PDR. The second aim is to analyze the behavior of partial redundancy by modifying the stochastic and functional model of the Kalman filter. The equations relating the observations to the orientation are condition equations, which do not exhibit the typical structure of the Gauss-Markov model (“GMM”), wherein the observations are linear and can be formulated as functions of the states. To calculate and analyze the partial redundancy of the observations from smartphone-sensors used in PDR, the system equation and the measurement equation of a Kalman filter as well as the redundancy matrix need to be derived in the Gauss-Helmert model (“GHM”). These derivations are introduced in this article and lead to a novel Kalman filter structure based on condition equations, enabling reliability assessment of each observation.

## 1. Introduction

Determining the orientation in pedestrian navigation with geometric-based approaches is an essential step for positioning. There are several possibilities to mathematically parameterize the orientation. In [1] three possibilities are given: the direction-cosine-matrix, Euler angles and quaternions. Discussion on the advantages and disadvantages of these concepts can be found in [2]. In this article the Euler angles—roll, pitch and yaw—are used as it is the yaw angle that is especially necessary for calculating the 2D-Position in pedestrian navigation approaches like Pedestrian Dead Reckoning (“PDR”). The orientation can be calculated directly by the use of an accelerometer and a magnetometer respectively a gyroscope, which are nowadays integrated in most of the available smartphones. Each of these sensors is subject to specific systematic effects [3], which have to be considered in detail when integrating low-cost sensors in smartphones. These systematic deviations clearly can have a noticeable impact on the resulting position of PDR, which makes determining the orientation the critical part.

Several approaches exist to fuse and improve the calculated orientation from the individual sensors. In [4] linear combinations are used, to calculate the yaw angle of the individual results from magnetometer and gyroscope. Using a Kalman filter to fuse accelerometer, magnetometer and gyroscope measurements [5,6,7] is the most common approach. Fusing these sensors offers the opportunity to detect and correct for systematic deviations in the observations. In [8] a Kalman filter is developed which uses the observed gravity and Earth magnetic field as well as the orientation quaternion which are propagated by means of the gyroscope. If static acceleration or magnetic flux is detected, systematic sensor deviations can be determined. In [9] or [10] the gyroscope is used to detect when the user is turning. If the user walks straight, systematic sensor deviations are derived. The same approach is used in [11] to smooth the resulting trajectory. A method to minimize systematic effects in acceleration measurements resulting from the steps of pedestrians by adapting the measurement noise can be found in [2]. Using additional data can improve the ability to detect systematic effects. One possibility to retrieve additional position and attitude information is to use pictures from the smartphone camera [12]. Also building plans can be used to support the calculation of the orientation [13]. The previously mentioned approaches only use observations from one device. Especially in pedestrian navigation, much data is produced from many different smartphones, which can be used for positioning in various ways. This crowd-sourced data is mainly used to support Wi-Fi fingerprinting [14,15] by extending and updating radio maps [16,17,18]. In the context of PDR, in [19] for example, trajectories from multiple users are used to minimize the influence of magnetic perturbations inside buildings.

Combining positions from signal strength observations with PDR is done, for example, in [20]—with an adaptive Kalman filter—or in [21]—with a particle filter–to improve positioning accuracy. Such signal strength observations or “Received Signal Strength” (RSS) can be used in geometric-based approaches or in feature-based approaches and require external infrastructure. Adding such positioning techniques—for example Wi-Fi [22,23], “radio-frequency identification” (RFID) [24] or artificial magnetic fields [25]—will increase the redundancy and minimize the influence of systematic effects. Geometric- and feature-based approaches are combined in [26], where also measures are derived to quantify accuracy independently from the distribution of the observations.

This paper focuses on determining the orientation for PDR with accelerometer, magnetometer and gyroscope in a Kalman filter. The focus is on analyzing the reliability of the observations from smartphone-sensors, which are non-linearly included in condition equations. The Kalman filter is commonly used to estimate states in a Gauss-Markov model (“GMM”—model with observation equations), wherein the observations of the measurement equation are linear and can be formulated as functions of the states [27,28]. However, the equations for the orientation determination in PDR are condition equations, containing implicit relations between states and observations. Thus, reliability analysis based on condition equations, necessitates a new derivation of the system equation and the measurement equation of a Kalman filter as well as the redundancy matrix in the Gauss-Helmert model (“GHM”—model with condition equations). The formulation of the system equation in the GHM can be found in [29,30], wherein the measurement equation and corresponding redundancy matrix are still assumed to satisfy the GMM. Hence, an important novelty of this article is the derivation of the whole Kalman filter structure in the GHM, enabling the possibility to calculate reliability measures for observations which are non-linearly included in the condition equations. 

Section 2 contains the explanation of the existing approach and subsequent problem description of reliability, as well as the derivation of the update-equations and redundancy matrix of the reformulated Kalman filter. The data of a measured trajectory will be used to estimate the Euler angles as well as the reliability measures. These results are presented in Section 3 whereas Section 4 concludes this contribution.

## 2. Orientation Determination

### 2.1. Existing Approach

In the Kalman filter used for orientation determination in this article, the state parameters are the Euler angles roll *ϕ*, pitch *θ* and yaw *ψ*. Based on the assumption that roll and pitch are constant while the user is walking, they are predicted at actual epoch *k* with a random-walk model (1) and (2). The yaw angle is predicted with two different system equations, whereby (3) will be used if the user is walking straight and (4) will be used at detected turns. In the second case the observed turn rates from gyroscope *g_y,k_* and *g_z,k_* are included as control quantities summed in the vector ***u**_k_*. By doing this, the predicted yaw should immediately follow the user’s turn. The noise components of the state parameters are *c_k−*1*,ϕ_, c_k−*1*,θ_, c_k−*1*,ψ_* and Δ*t* is the time interval between two consecutive Kalman updates:

(1) $$f_{1}:\varphi_{k}=\varphi_{k-1}+c_{k-1,\varphi }$$

(2) $$f_{2}:\theta_{k}=\theta_{k-1}+c_{k-1,\theta }$$

(3) $$f_{3}:\psi_{k}=\psi_{k-1}+c_{k-1,\psi }$$

(4) $$f_{3}:\psi_{k}=\psi_{k-1}+\frac{\Delta t}{\cos\theta_{k-1}}\left(g_{y,k}\sin\varphi_{k-1}+g_{z,k}\cos\varphi_{k-1}\right)+c_{k-1,\psi }$$

Turn detection is done by applying a statistical test on the filter innovations of the yaw angle *d_ψ,i_* [29,31]. The null hypothesis *H*_0_ of this statistical test says that the vector ***d**_ψ_* containing the filter innovations *d_ψ,i_* from the last *n* epochs is equal to the zero vector (5), whereas the alternate hypothesis *H_A_* states that ***d**_ψ_* is significantly different to the zero vector (6). If the test value exceeds the corresponding quantile of the chi-square distribution (7), (4) will be used to predict the yaw angle. ***D**_ψ_* is the variance-covariance matrix (VCM) of the innovation vector ***d**_ψ_* and only contains variances on the diagonal belonging to the corresponding *d_ψ,i_*. This is a simplification, as auto-covariances may be present. The use of random-walk (3) results in smoother trajectories in sections when the user walks straight. By neglecting the observations from gyroscope in the random-walk model, the influence of systematic sensor deviations (gyro-drift) on the filter result is minimized:

(5) $$H_{0}:E(d_{\psi })=E\left({\left[\begin{array}{cccc} d_{\psi ,1} & d_{\psi ,2} & \cdots & d_{\psi ,n} \end{array}\right]}^{T}\right)=0$$

(6) $$H_{A}:E(d_{\psi })\neq 0$$

(7) $$P\{d_{\psi }^{T}D_{\psi }^{-1}d_{\psi }\leq \chi_{n;1-\alpha }^{2}|H_{0}\}=1-\alpha$$

For the update equations also the VCM of the predicted state is needed (8):

(8) $$\sum_{\bar{x}\bar{x},k}=T_{k,k-1}\sum_{\hat{x}\hat{x},k-1}T_{k,k-1}^{T}+U_{k,k-1}\sum_{uu,k}U_{k,k-1}^{T}+C_{k,k-1}\sum_{cc,k}C_{k,k-1}^{T}$$

Therein, $\Sigma_{ii,k}$—with the index *i* = ${\hat{x}}_{k-1}$, *u*, *c*—are the VCMs of the corresponding observation groups in epoch *k*. ***T**_k,k_*_−1_ is the state transition matrix, ***U**_k,k_*_−1_ the control matrix and ***C**_k,k_*_−1_ is the noise matrix, each referred to epoch *k*. These system matrices are Jacobi matrices and in general contain the derivatives of the system equations with respect to the corresponding observation group [27,28,29]. Equations (9)–(11) show the system matrices for the approach presented in this section where ***E*** is the identity matrix.

(9) $$T_{k,k-1}=\left[\begin{array}{ccc} 1 & 0 & 0\\ 0 & 1 & 0\\ \frac{\partial f_{3}}{\partial \varphi_{k}} & \frac{\partial f_{3}}{\partial \theta_{k}} & 1 \end{array}\right]$$

(10) $$U_{k,k-1}=\left[\begin{array}{ccc} 0 & 0 & 0\\ 0 & 0 & 0\\ 0 & \frac{\partial f_{3}}{\partial g_{y,k}} & \frac{\partial f_{3}}{\partial g_{z,k}} \end{array}\right]$$

(11) $$C_{k,k-1}=\Delta t\cdot E_{3\times 3}$$

The observations in the measurement equation are directly observed Euler angles, as also shown in [32]. Therefore, the design matrix ***A**_m,k_* of the Kalman filter equals the identity matrix. They are calculated outside of the Kalman filter ((12)–(14)) by using observed accelerations *a_x,k_*, *a_y,k_*, *a_z,k_* and magnetic flux densities *m_x,k_*, *m_y,k_*, *m_z,k_* [33]. The accelerations are filtered in a separate Kalman filter to remove high-frequency components due to the movement of the user [2]:

(12) $$f_{4}:\varphi_{k}=\tan^{-1}\left(\frac{a_{y,k}}{a_{z,k}}\right)$$

(13) $$f_{5}:\theta_{k}=\tan^{-1}\left(\frac{-a_{x,k}}{a_{y,k}\sin\left(\varphi_{k}\right)+a_{z,k}\cos\left(\varphi_{k}\right)}\right)$$

(14) $$f_{6}:\psi_{k}=\tan^{-1}\left(\frac{m_{z,k}\sin\left(\varphi_{k}\right)-m_{y,k}\cos\left(\varphi_{k}\right)}{m_{x,k}\cos\left(\theta_{k}\right)+m_{y,k}\sin\left(\theta_{k}\right)\sin\left(\varphi_{k}\right)+m_{z,k}\sin\left(\theta_{k}\right)\cos\left(\varphi_{k}\right)}\right)$$

The yaw angle calculated with (14) can be subject to magnetic perturbations, especially inside buildings. The redundant determination of the Euler angles in the Kalman filter by using the system equation and the measurement equation dampens the influence of magnetic perturbations. Additionally, the standard deviation of the magnetometer measurements *σ_m_* will be increased if the magnitude of the measured magnetic field $\left\|m_{k}\right\|$ is not stable (15), leading to an adaptive standard deviation *σ_m,k_* for each Kalman filter epoch. As the geomagnetic field should be constant related to the dimensions of a building, it will be assumed that magnetic perturbations are present if the measured magnitude changes:

(15) $$\sigma_{m,k}=\sigma_{m}+\left|\left\|m_{k}\right\|-\left\|m_{k-1}\right\|\right|$$

Using the covariance propagation law, the VCM belonging to the directly observed Euler angles $\Sigma_{\varphi \theta \psi ,k}$ is derived (16), wherein ***H**_k_* is a Jacobi matrix containing the derivatives of (12)–(14) with respect to the accelerometer and magnetometer measurements.

(16) $$\begin{array}{l} \sum_{\varphi \theta \psi ,k}=H_{k}\left[\begin{array}{cc} \sum_{aa,k} & 0_{3\times 3}\\ 0_{3\times 3} & \sigma_{m,k}E_{3\times 3} \end{array}\right]H_{k}^{T}\\ H_{k}=\left[\begin{array}{cccccc} 0 & \frac{\partial f_{4}}{\partial a_{y,k}} & \frac{\partial f_{4}}{\partial a_{z,k}} & 0 & 0 & 0\\ \frac{\partial f_{5}}{\partial a_{x,k}} & \frac{\partial f_{5}}{\partial a_{y,k}} & \frac{\partial f_{5}}{\partial a_{z,k}} & 0 & 0 & 0\\ \frac{\partial f_{5}}{\partial a_{x,k}} & \frac{\partial f_{5}}{\partial a_{y,k}} & \frac{\partial f_{5}}{\partial a_{z,k}} & \frac{\partial f_{5}}{\partial m_{x,k}} & \frac{\partial f_{5}}{\partial m_{y,k}} & \frac{\partial f_{5}}{\partial m_{z,k}} \end{array}\right] \end{array}$$

### 2.2. Problem Description

**Figure 1:**
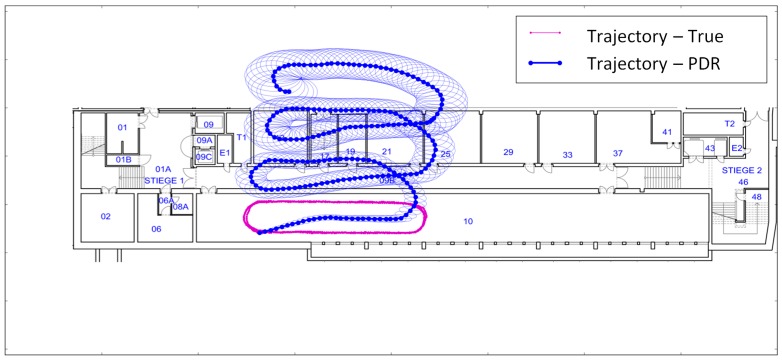
PDR Trajectory. Ground truth in magenta, estimated steps in blue with 95% confidence ellipses.

**Figure 2:**
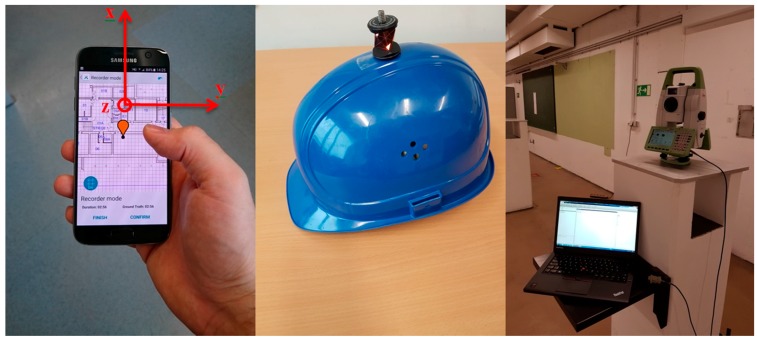
Used sensors and measurement setup: Samsung Galaxy S7 (Samsung, Seoul, Korea), smartphone running indoo.rs Mobile Toolkit^TM^ to collect sensor observations (**left**). Helmet with 360°-mini-prism (**middle**) and Leica TS16 tracking the user (**right**).

To determine the user’s position, the step length is also estimated and will be used with fixed variance in PDR. Figure 1 shows a trajectory calculated with PDR, whereby the yaw is estimated with the Kalman filter mentioned in Section 2.1. Additionally, the 95% confidence ellipses as well as the reference trajectory are part of Figure 1. This measured trajectory will also be used to analyze the partial redundancy in Section 3. During the measurements, the user held the smartphone in portrait mode (Figure 2 left, *ϕ_k_* ~ 0°). The reference trajectory is calculated from the measurements of the TS16 total station (Leica Geosystems, Heerbrugg, Switzerland) which is tracking the user by the help of a 360°-mini-prism on a helmet (Figure 2 right).

In PDR, the yaw angle is responsible for the shape of the trajectory and the step length for the scale. Hence in Figure 1, the estimated orientation causes the deviations between the estimated and the reference trajectory. As these deviations are not captured by the confidence ellipses—which are a measure for the precision [34]—there are two possible reasons for their appearance: systematic deviations in the observed data or non-Gaussian distributed data. Because of the obvious systematics in the estimated trajectory, it is assumed that systematic deviations are present in the observed data.

Reliability theory deals with the detection of large systematic deviations (inner reliability) and their effect on the estimated quantities (outer reliability) [35,36]. To identify the measurements responsible for the systematic deviations of the orientation between estimated and reference trajectory, the inner reliability is used. Here the partial redundancies *r_i_* play a key role. According to [29,37,38], all quantities with stochastic information can be treated as observations.

Observations related to the system equation are the estimated state from the previous epoch ${\hat{x}}_{k-1}$, the control variables ***u*** and the noise variables ***c***. The observations of the measurement equation are summed in the vector $l_{m}$, where *m* labels quantities of the measurement equation. With (17)–(20) the partial redundancy can be calculated for the previously mentioned observations according to [29]:

(17) $$\sum_{j=1}^{n_{x}}r_{{\hat{x}}_{k-1},k,j}=\sum_{j=1}^{n_{x}}e_{j}^{T}\sum_{\hat{x}\hat{x},k-1}T_{k,k-1}^{T}A_{m,k}^{T}D_{k}^{-1}A_{m,k}T_{k,k-1}e_{j}$$

(18) $$\sum_{j=1}^{n_{u}}r_{u,k,j}=\sum_{j=1}^{n_{u}}e_{j}^{T}\sum_{uu,k}U_{k,k-1}^{T}A_{m,k}^{T}D_{k}^{-1}A_{m,k}U_{k,k-1}e_{j}$$

(19) $$\sum_{j=1}^{n_{w}}r_{c,k,j}=\sum_{j=1}^{n_{w}}e_{j}^{T}\sum_{cc,k}C_{k,k-1}^{T}A_{m,k}^{T}D_{k}^{-1}A_{m,k}C_{k,k-1}e_{j}$$

(20) $$\sum_{j=1}^{n_{l}}r_{l_{m},k,j}=\sum_{j=1}^{n_{l}}e_{j}^{T}\sum_{ll,m,k}D_{k}^{-1}e_{j}$$

Therein, *n_i_* is the number of observations in each observation group and ***D**_k_* is the VCM of the filter innovation [27,28,29]. ***e**_j_* is the unity vector to select the corresponding *r_i_* respectively diagonal element. In the above formulation of the Kalman filter, the *r_i_* can only be calculated for the directly observed angles in the measurement equation but not for the original observations from accelerometer and magnetometer due to the structure of (12)–(14).

### 2.3. Kalman Filter in the Gauss-Helmert Model

In the GMM, the true observations $\tilde{l}$ can be modeled as a function of the true parameters $\tilde{x}$—which also holds for the estimated observations $\hat{l}$ and estimated parameters $\hat{x}$ (21). As mentioned above, the measurement Equations (12)–(14) have another structure, which matches the GHM [39] shown in (22). Hence, the update equations of the Kalman filter have to be derived in the GHM, to directly use the observations from smartphone-sensors. Afterwards, the corresponding *r_i_* need to be derived for this case. In [30] the GHM is also used to estimate variance components for the system noise. Though, there is still the assumption of using the GMM in the measurement equation and the results cannot be used in this article.

(21) $$\tilde{l}-f\left(\tilde{x}\right)=\hat{l}-f\left(\hat{x}\right)=0$$

(22) $$f\left(\tilde{l},\tilde{x}\right)=f\left(\hat{l},\hat{x}\right)=0$$

As the functional relations (22) are non-linear in general—and especially in this article—they have to be linearized. Neither the real observations and parameters, nor their estimated values are known a priori. Hence, to do Taylor linearization, approximate values $l_{0}$ and $x_{0}$ have to be used which also have to satisfy the non-linear relations [34]. In least-squares, especially $x_{0}$ is assumed to be non-stochastic. According to [40], $l_{0}$ can be replaced by the observed data l after linearization, which results in the linearized, functional model (23):

(23) $$f(l,x_{0})+A(\hat{x}-x_{0})+B(\hat{l}-l)=0$$

The first term corresponds to the misclosure vector **w**, the bracketed expression in the second term equals the stochastic additions $\Delta \hat{x}$ to the approximate parameters $x_{0}$ and the bracketed expression in the third term equals the residuals v. Formula (23) is only valid for the first iteration of least-squares estimation, because linearization of the functional model necessitates an iterative approach. In the subsequent iterations, the functional model is always linearized at the previously estimated observations and parameters [40]. Formulas (24)–(27) show, how the searched quantities with corresponding VCM are calculated in the GHM in the context of least-squares [41]:

(24) $$\sum_{\Delta \hat{x}\Delta \hat{x}}={\left(A^{T}{\left(B\sum_{ll}B^{T}\right)}^{-1}A\right)}^{-1}$$

(25) $$\Delta \hat{x}=-\sum_{\Delta \hat{x}\Delta \hat{x}}A^{T}{\left(B\sum_{ll}B^{T}\right)}^{-1}w$$

(26) $$v=-\sum_{ll}B^{T}{\left(B\sum_{ll}B^{T}\right)}^{-1}\left(E-A\sum_{\Delta \hat{x}\Delta \hat{x}}A^{T}{\left(B\sum_{ll}B^{T}\right)}^{-1}\right)w$$

(27) $$\sum_{vv}=\sum_{ll}B^{T}{\left(B\sum_{ll}B^{T}\right)}^{-1}\left(E-A\sum_{\Delta \hat{x}\Delta \hat{x}}A^{T}{\left(B\sum_{ll}B^{T}\right)}^{-1}\right)B\sum_{ll}$$

***B*** is the observation matrix, $\Sigma_{\Delta \hat{x}\Delta \hat{x}}$ is the VCM of the estimated additions, $\Sigma_{ll}$ the VCM of the observations and $\Sigma_{\mathrm{vv}}$ is the VCM of the residuals. It is important that ***A*** and ***B*** are column-regular matrices. Otherwise the inverse matrices cannot be calculated. To avoid singular matrices, the functional relations have to be chosen, such that there will be no linearly dependent columns in these matrices.

The Kalman filter is based on sequential least-squares with an additional state transition model. The state transition model respectively system equation is described in general by non-linear, stochastic, vector-matrix differential equations. We assume, that such differential equations have the form shown in (28) after linearization, which is also basis for deriving the system equation in [27,28,29]:

(28) $$\dot{x}(t)=Fx(t)+Lu(t)+Gc(t)$$

*t* is the continuous time variable. The system matrix ***F***, control-input matrix ***L*** and noise-input matrix ***G*** are assumed to be time-invariant. Solving such differential equations in the state space—also shown in [27,28,29]—unambiguously defines the state parameters at time *k* and gives the approximate formulation (29):

(29) $$x_{k}\approx T_{k,k-1}x_{k}+U_{k,k-1}u_{k}+C_{k,k-1}c_{k}$$

As mentioned in [27], the predicted parameter vector ${\bar{x}}_{k}$ should be calculated from the original set of functions (30) in the non-linear case to avoid linearization errors. Equation (29) is necessary for calculating the VCM of ${\bar{x}}_{k}$ with the covariance propagation law (8).

(30) $${\bar{x}}_{k}=f({\hat{x}}_{k-1},u_{k},c_{k})$$

The system can also be described by several sub-systems which yields in an over-determined system equation. This will be dealt with in the next section, where one yaw angle will be estimated from multiple trajectory data. Thus, the system equation decomposes into two sets of equations. The first set unambiguously defines the predicted parameters by using ${\hat{x}}_{k-1}$, ***u**_k_* and ***c**_k_* which is already shown in (29) respectively (30). This set of equations will be—analogue to [29]—formulated in the GHM, such that it contains the residuals belonging to ${\hat{x}}_{k-1}$, ***u**_k_* and ***c**_k_* (31). The derivation of (31) can be found in the Appendix A:

(31) $${\bar{x}}_{k}-{\hat{x}}_{k}+v_{\bar{x},k}=-E\Delta {\hat{x}}_{k}+\left[\begin{array}{ccc} T_{k,k-1} & U_{k,k-1} & C_{k,k-1} \end{array}\right]\left[\begin{array}{c} v_{{\hat{x}}_{k-1},k}\\ v_{u,k}\\ v_{c,k} \end{array}\right]=0$$

The second set consists of condition equations, having the same structure like (23). These condition equations contain the same quantities like the first set of equations and can therefore be formulated as shown in (32). w^*^_s,k_ are the misclosures, arising because of the overdetermined system equation respectively condition equations. A^*^_s,k_ is the design matrix and T^*^_k.k__−1_, U^*^_k,k__−1_ and C^*^_k,k__−1_ are the observation matrices which belong to the condition equations of the system equation:

(32) $$w_{s,k}^{*}+A_{s,k}^{*}\Delta {\hat{x}}_{k}+\left[\begin{array}{ccc} T_{k,k-1}^{*} & U_{k,k-1}^{*} & C_{k,k-1}^{*} \end{array}\right]\left[\begin{array}{c} v_{{\hat{x}}_{k-1},k}\\ v_{u,k}\\ v_{c,k} \end{array}\right]=0$$

The functional model of the measurement equation has the structure (23). Fusing the system equation and the measurement equation leads to the functional model of a Kalman filter formulated in the GHM with additional condition equations in the system Equation (33):

(33) $$\left[\begin{array}{c} \begin{array}{c} 0\\ w_{s,k}^{*} \end{array}\\ w_{m,k} \end{array}\right]+\left[\begin{array}{c} \begin{array}{c} -E\\ A_{s,k}^{*} \end{array}\\ A_{m,k} \end{array}\right]\Delta {\hat{x}}_{k}+\left[\begin{array}{c} T_{k,k-1}\\ T_{k,k-1}^{*}\\ 0 \end{array}\begin{array}{c} U_{k,k-1}\\ U_{k,k-1}^{*}\\ 0 \end{array}\begin{array}{c} C_{k,k-1}\\ C_{k,k-1}^{*}\\ 0 \end{array}\begin{array}{c} 0\\ 0\\ B_{m,k} \end{array}\right]\left[\begin{array}{c} v_{{\hat{x}}_{k-1},k}\\ v_{u,k}\\ v_{c,k}\\ v_{l_{m},k} \end{array}\right]=\underset{w}{\underset{︸}{\left[\begin{array}{c} w_{s,k}\\ w_{m,k} \end{array}\right]}}+\underset{A}{\underset{︸}{\left[\begin{array}{c} A_{s,k}\\ A_{m,k} \end{array}\right]}}\Delta {\hat{x}}_{k}+\underset{B}{\underset{︸}{\left[\begin{array}{cc} B_{s,k,k-1} & 0\\ 0 & B_{m,k} \end{array}\right]}}\underset{v}{\underset{︸}{\left[\begin{array}{c} v_{l_{s},k}\\ v_{l_{m},k} \end{array}\right]}}=0$$

Now the formulas of the GHM in least-squares can be applied and the parameters with corresponding VCM can be calculated. Equation (34) shows the VCM of the estimated parameters, which is derived by inserting ***A*** and ***B*** into (24):

(34) $$\sum_{\hat{x}\hat{x},k}={\left(A_{s,k}^{T}{\left(B_{s,k,k-1}\sum_{ll,s,k}B_{s,k,k-1}^{T}\right)}^{-1}A_{s,k}+A_{m,k}^{T}{\left(B_{m,k}\sum_{ll,m,k}B_{m,k}^{T}\right)}^{-1}A_{m,k}\right)}^{-1}={\left(\sum_{\hat{x}\hat{x},s,k}^{-1}+\sum_{\hat{x}\hat{x},m,k}^{-1}\right)}^{-1}$$

$\Sigma_{\hat{x}\hat{x},s,k}$ is the VCM of the parameters which would be the result of a Kalman filter only considering the system equation. Similarly, $\Sigma_{\hat{x}\hat{x},m,k}$ is the VCM of the parameters if only the measurement equation would be considered in the Kalman filter. $\Sigma_{\hat{x}\hat{x},k}$ is the inverse of the sum of these two matrices. To further process (34), the Woodbury formula for matrix inversion—according to [42]—is applied (35), where ***M***, ***N***, ***O*** and ***P*** are arbitrary matrices and not related to the derivations made in this section:

(35) $${\left(M+NOP\right)}^{-1}=M^{-1}-M^{-1}N{\left(O^{-1}+PM^{-1}N\right)}^{-1}PM^{-1}$$

Depending on which term of the sum in (34) is chosen to be the matrix ***M*** in the Woodbury formula (35), results will show two equivalent representations (36) and (37) of the VCM of the estimated parameters. It has to be mentioned, that this VCM corresponds to ${\hat{x}}_{k}$ and not to $\Delta {\hat{x}}_{k}$. The reason is that $x_{0}$ equals ${\bar{x}}_{k}$, which is stochastic and its stochastic information is implicitly integrated by adding its calculation to the functional model ((31) and (33)). Whereas in least-squares $x_{0}$ is non-stochastic and therefore $\Sigma_{\hat{x}\hat{x}}$ equals $\Sigma_{\Delta \hat{x}\Delta \hat{x}}$.

(36) $$\sum_{\hat{x}\hat{x},k}=\left(E-\sum_{\hat{x}\hat{x},s,k}A_{m,k}^{T}{\left(B_{m,k}\sum_{ll,m,k}B_{m,k}^{T}+A_{m,k}\sum_{\hat{x}\hat{x},s,k}A_{m,k}^{T}\right)}^{-1}A_{m,k}\right)\sum_{\hat{x}\hat{x},s,k}$$

(37) $$\sum_{\hat{x}\hat{x},k}=\left(E-\sum_{\hat{x}\hat{x},m,k}A_{s,k}^{T}{\left(B_{s,k,k-1}\sum_{ll,s,k}B_{s,k,k-1}^{T}+A_{s,k}\sum_{\hat{x}\hat{x},m,k}A_{s,k}^{T}\right)}^{-1}A_{s,k}\right)\sum_{\hat{x}\hat{x},m,k}$$

By comparing (36) with the update equation for the VCM of the estimated parameters in the GMM (see [29] or [27]), the VCM of the filter innovation ***D**_m,k_* and the gain matrix ***K**_m,k_*—belonging to the measurement equation—can be found ((39) and (41)). In the same manner, the VCM of the filter innovation ***D**_s,k_* of and the gain matrix ***K**_s,k_* belonging to the system equation are derived ((38) and (40)):

(38) $$D_{s,k}=B_{s,k,k-1}\sum_{ll,s,k}B_{s,k,k-1}^{T}+A_{s,k}\sum_{\hat{x}\hat{x},m,k}A_{s,k}^{T}$$

(39) $$D_{m,k}=B_{m,k}\sum_{ll,m,k}B_{m,k}^{T}+A_{m,k}\sum_{\hat{x}\hat{x},s,k}A_{m,k}^{T}$$

(40) $$K_{s,k}=\sum_{\hat{x}\hat{x},m,k}A_{s,k}^{T}D_{s,k}^{-1}$$

(41) $$K_{m,k}=\sum_{\hat{x}\hat{x},s,k}A_{m,k}^{T}D_{m,k}^{-1}$$

The estimated additions to the approximate parameters are derived by using (25) and (33). Formula (42) shows these additions, where the results from the previous paragraph are already considered. Hence, it includes corrections respectively updates for the system equation as well as for the measurement equation:

(42) $$\Delta {\hat{x}}_{k}=-K_{s,k}w_{s,k}-K_{m,k}w_{m,k}=-\left[\begin{array}{cc} K_{s,k} & K_{m,k} \end{array}\right]\left[\begin{array}{c} w_{s,k}\\ w_{m,k} \end{array}\right]$$

If the system equation is not overdetermined, (32) will not be used in the functional model (33), which leads to simplifications in the update equations. The classical Kalman filter update equations as well as the two GHM variants are summarized in Table 1.

Approaches whose aim is to detect systematic deviations in the observations respectively to quantify the inner reliability in the GMM, are often based on the disturbed residuals $\bar{v}$ [43]. The error-term ∇v in (43) is caused by the systematic deviations in the observations, summed in the error-vector ∇l:

(43) $$\bar{v}=v+\nabla v$$

*r_i_* is the factor which specifies how an observation deviation $\nabla l_{i}$ influences the corresponding residual $v_{i}$ (44). Hence, high *r_i_* are desirable to detect systematic deviations [29]:

(44) $$\nabla v_{i}=-r_{i}\nabla l_{i}$$

Transferring these thoughts to the GHM, (26) has to be used to derive ***R***, containing the *r_i_* on its diagonal. ***R*** is an idempotent matrix, whose trace equals the overall redundancy of the estimation problem [34]. Formula (26) only contains the misclosures **w**, which can be linearized by ***Bl*** according to [34]. If ∇l is taken into account, results show the disturbed model (45):

(45) $$\bar{v}=-\sum_{ll}B^{T}{\left(B\sum_{ll}B^{T}\right)}^{-1}\left(E-A\sum_{\Delta \hat{x}\Delta \hat{x}}A^{T}{\left(B\sum_{ll}B^{T}\right)}^{-1}\right)B\left(l+\nabla l\right)$$

Thus, there is a direct relation between observations and residuals and the redundancy matrix for the GHM is now available (46):

(46) $$R=\sum_{ll}B^{T}{\left(B\sum_{ll}B^{T}\right)}^{-1}\left(E-A\sum_{\hat{x}\hat{x}}A^{T}{\left(B\sum_{ll}B^{T}\right)}^{-1}\right)B$$

By using (27), (46) equals the matrix product $\Sigma_{\mathrm{vv}}\Sigma_{ll}^{-1}$, whereby the analogy to the GMM is stated again [34]. The proof that the trace of ***R*** equals the overall redundancy can be found in Appendix B.

## 3. Results

**Figure 3:**
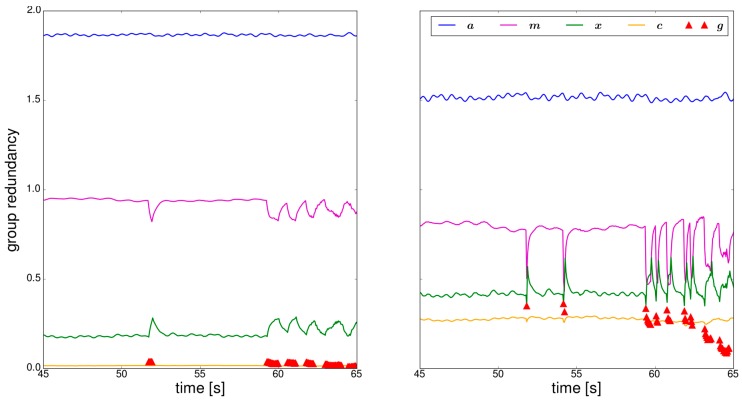
Redundancies of the observation groups used in the Kalman filter. (**Left**) Calculation with the original standard deviations; (**Right**) Calculation with adapted standard deviations.

In this section the results of the trajectory shown in Section 2.2. will be analyzed by applying the simplified GHM (Table 1) on the approach described in Section 2.1. In a first step, the influence of the observation groups—accelerometer ***a***, magnetometer ***m***, estimated parameters of the previous epoch ***x***, system noise ***c*** and gyroscope ***g***—on the estimated orientation will be assessed by means of the *r_i_*. Figure 3 shows a representative section of the calculated group redundancies for two different specifications of the sensor- and system noise standard deviations.

For the beginning the focus lies on the left part of Figure 3, where the original standard deviations are used (Table 2). These are derived for each observation in the trajectory parts where the user walked straight. Using these standard deviations leads to a small partial redundancy of the observation groups (***x***, ***c***, ***g***) of the system equation in comparison to the ones of the measurement equation (***a***, ***m***). This means that the estimated orientation mainly relies on the system equation. The reason for the high weight of the system equation is that the resulting trajectory should be smoothed [32]. The problem is that if the model assumptions made in the system equation do not capture the reality, the resulting deviations have high influence on the estimated orientation. There are mainly two possibilities to intervene in the partial redundancy respectively the inner reliability, which will be covered in the next two sections.

### 3.1. Adaption of the Stochastic Model

Adaption of the stochastic model means to change the standard deviations of the different observations. To align the group redundancies of the observation groups (Figure 3 left), the standard deviations of the sensors and system noise are changed until an improvement is visible. This leads to the group redundancies in the right part of Figure 3, where the adapted standard deviations of Table 2 were used. The weight of the system equation in comparison to the measurement equation is now reduced, whereas the *r_i_* of the gyroscope and the system noise are now clearly higher. The *r_i_* of the magnetometer get close to the ones of the gyroscope if it is used when the user is turning, which yields in good mutual controllability.

**Figure 4:**
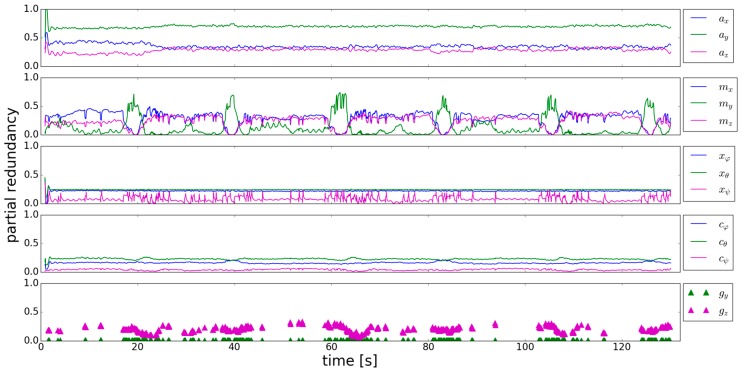
Partial redundancy of the accelerometer, magnetometer, previously estimated parameter, system noise and gyroscope (**top** to **bottom**).

To identify the critical observations, the *r_i_* of the individual observations are analyzed. Figure 4 shows, that the *r_i_* of the accelerometer are continuously higher than 0.2, which means that controllability is sufficient. Calculating the thresholds from which deviations can be—statistically justified—detected (according to [29]), gives ~1.4 m/s^2^ for the y-component of the accelerometer and ~2 m/s^2^ for the x- and z-component. The user’s motion causes systematic deviations which exceeds these thresholds. Hence, accelerometer observations which should not be used for calculating pitch and roll can be detected.

The *r_i_* of the magnetometer show a remarkable, alternating behavior. In the trajectory parts where the user walks straight, the *r_i_* of *m_y_* are close to zero. Whereas in parts where the user turns, controllability of *m_x_* and *m_z_* is bad. If there are systematic deviations in these observations, they cannot be detected and have a high influence on the estimated orientation angles. The *r_i_* of *x_ϕ_* and *x_θ_* are again continuously higher than 0.2 and therefore sufficiently controlled. The same findings hold for *c_ϕ_* and *c_θ_*, whereas *c_ψ_* is totally uncontrolled. Generally the *r_i_* of the previously estimated parameters and the system noise behave in a similar manner. *g_y_* is also uncontrolled as its *r_i_* is very close to zero. The *r_i_* of *g_z_* are again higher than 0.2. The behavior of the *r_i_* of the previously estimated yaw angle is interesting, as they go up to 0.25 if the gyroscope measurements are used in the system equation. If the gyroscope is not used in the following epochs the *r_i_* decrease.

From the analysis of Figure 4 it can be concluded that there are uncontrollable observations in this approach, even if the standard deviations are adapted. To better understand the behavior of the *r_i_*, the influence of the change of the standard deviation *σ_i_* of one observation on the partial redundancy of the other observations is analyzed. This analysis should support an aimed change of the standard deviations to improve the *r_i_*. Table 3 shows how the *r_i_* change, if the standard deviation of one observation is multiplied with the factor 10 (left sign in Table 3) respectively 0.1 (right sign in Table 3). The standard deviation of all the observations was varied, except for the ones of the previously estimated parameters, as they are a direct result of the Kalman filter.

The grey shaded cells show the influence of a change of *σ_i_* of one observation on the corresponding *r_i_*. If *σ_i_* is increased, the weight of the observation will be less in state estimation. Hence the corresponding *r_i_* should also be increased. A reduction of *σ_i_* should cause a smaller *r_i_* on the contrary. This is the case for most of the observations, except for *g_y_*. The reason therefore is that its *r_i_* are already close to zero and a reduction of *σ_i_* has no effect.

Green shaded cells show a direct functional relation of the corresponding observations (see (1)–(4) and (12)–(14)). The change of *σ_i_* of one observation influences also the partial redundancy of other observations. A raise of one *σ_i_* should cause a reduction of partial redundancy of other observations, as they get more weight in the state estimation. A reduction of one *σ_i_* should raise the partial redundancy of other observations on the contrary. Though, the change of *σ_i_* of *c_ϕ_* and *c_θ_*, show another behavior. By increasing as well as decreasing *σ_c-ϕ_* and *σ_c-θ_*, the *r_x-ϕ_* and *r_x-θ_* are reduced. A raise of *σ_i_* of *g_z_* respectively of *c_ψ_* causes also a raise of the *r_i_* of the previously estimated yaw angle.

A relation which is not expected from the system equations respectively the measurement equations, appears at the accelerometer measurements. *a_y_* is only related to *x_ϕ_* and *c_ϕ_*, whereas *a_x_* and *a_z_* are related to *x_θ_* and *c_θ_*. The change of *σ_i_* of one of the magnetometer measurements influences the *r_i_* of *x_ψ_*, *g_z_* and the other two magnetometer components. *m_y_* stands out, as a reduction of *σ_i_* positively influences the *r_i_* of *c_ϕ_* and *c_θ_* and the *r_i_* of *a_z_*. Hence, a reduction of *σ_i_* of *m_y_* would have the most positive influence on the *r_i_*. As the controllability of this observation is bad, a further reduction is not advisable.

Changing the stochastic model clearly has an impact on the inner reliability but it also has limitations. As shown in Table 3, *g_y_* as well as *c_ψ_* are not controlled by any of the other observations (i.e., changing the standard deviation of any other observation does not influence *r_g-y_* and *r_c-ψ_*). The only way left in the stochastic model is to increase the standard deviation of such observations. In this case it is very likely, that the raised standard deviations cover the systematic deviations which actually should be detected.

### 3.2. Adaption of the Functional Model

In general, the functional model can be adapted by changing respectively extending the system equations and the measurement equations. As seen in the previous section, the y-component of the gyroscope and the system noise of the yaw angle stay problematical observations, as their controllability cannot be improved by changing *σ_i_* of other observations. Through the example of the *r_i_* of the gyroscope, the functional influences should be analyzed. (47) and (48) show the formulas to calculate the *r_i_* of the gyroscope:

(47) $$r_{g-y}=\sin^{2}\varphi \cdot \frac{\sigma_{g-y}^{2}\cdot d_{33}^{*}\cdot \Delta t^{2}}{\cos^{2}\theta }$$

(48) $$r_{g-z}=\cos^{2}\varphi \cdot \frac{\sigma_{g-z}^{2}\cdot d_{33}^{*}\cdot \Delta t^{2}}{\cos^{2}\theta }$$

These equations are derived by evaluating (18), which is shown in Appendix C. *d*_33_* corresponds to the third diagonal element of the inverse of ***D***. The difference of (47) and (48) is the trigonometric function of the roll angle *ϕ* (*σ*^2^*_g-y_* and *σ*^2^*_g-z_* are assumed to be equal). As the roll angle is close to zero in the considered trajectory, also *r_g-y_* will be close to zero even when changing *σ_i_* of other observations (which influences *d*_33_*). Hence, the smartphone orientation during the trajectory measurements has a huge impact on the calculated *r_i_*—not only the ones from gyroscope, but also the ones from accelerometer and magnetometer ((12)–(14)). This seems reasonable if a closer look is taken on the quantities used to determine the smartphone orientation. The accelerometer should sense the gravity vector and the magnetometer the Earth magnetic field, which are both a vector quantity. The more sensor components sense these quantities, the better the mutual controllability should be. This should be the same for the gyroscope, which should sense the rotation of the user around the z-axis of the reference respectively navigation coordinate frame.

To check whether the assumptions made above are true, the recorded sensor data from the trajectory are rotated with the rotation matrix ***R*** given in (49):

(49) $$\begin{array}{l} R=R_{x}R_{y}R_{z}\\ R_{x}=\left[\begin{array}{ccc} 1 & 0 & 0\\ 0 & \cos\varphi & -\sin\varphi \\ 0 & \sin\varphi & \cos\varphi \end{array}\right],\;R_{y}=\left[\begin{array}{ccc} \cos\theta & 0 & \sin\theta \\ 0 & 1 & 0\\ -\sin\theta & 0 & \cos\theta \end{array}\right],\;R_{z}=\left[\begin{array}{ccc} \cos\psi & -\sin\psi & 0\\ \sin\psi & \cos\psi & 0\\ 0 & 0 & 1 \end{array}\right] \end{array}$$

**Figure 5:**
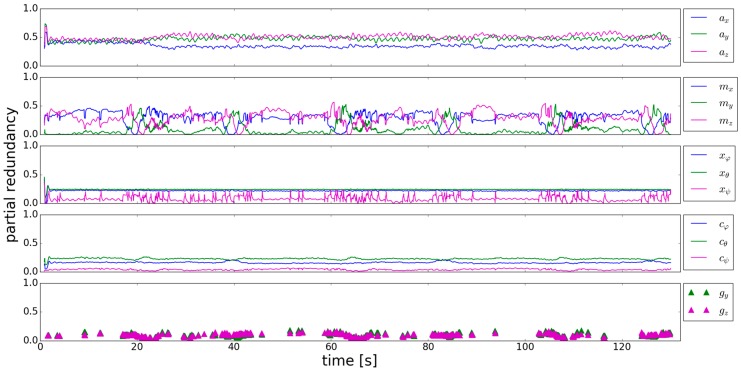
Partial redundancy of the rotated trajectory.

*ϕ* is chosen to be 45° and *θ* and *ψ* are 0°. Evaluating the rotated sensor data in the same Kalman filter as used above, gives the partial redundancies shown in Figure 5. The *r_i_* of the previously estimated parameters, the system noise as well as the x-components of the sensors are not affected by the rotated data, when comparing the results with Figure 4. The y- and z-components of the sensors are more balanced now, leading to a better mutual controllability. Especially the mean level of the *r_i_* of the gyroscope y-component are now approximately 0.1, which is clearly higher compared to Figure 4.

The idea of the following approach is to use multiple trajectory data, to determine the actual smartphone’s orientation. If one thinks of scenarios in crowded environments, it could be that multiple users have already taken the same path as the actual user does. If this data from the users who have taken the same path is stored, it could be used in a multiple trajectory data approach to determine the actual yaw angle, which equals the situation outlined in Section 2.3 where several sub-systems are used to determine the overall-system. Hence, the Gauss-Helmert Kalman filter which incorporates (32) in its functional model (33) has to be used now. In the case of orientation determination, the set of equations which unambiguously determine the state vector (31) are equal to (1), (2) and (3) respectively (4) for the actual trajectory. Now, for every additionally used trajectory, (1) and (2) have to be added to the unambiguous set of equations, as the smartphone’s orientation could be different (the only assumption is that there is the same path, e.g., the same yaw angle). Thus—if *N_tr_* is the number of used trajectories—*N_tr_* roll angles, *N_tr_* pitch angles and one yaw angle have to be estimated. If there is a turn detected in the actual trajectory, gyroscope measurements from multiple smartphones have to be processed. Hence, every additional trajectory contributes with a formula of type (4) to the system equation, which leads to *N_tr_*−1 condition Equations (32) in the system equation. The measurement equation consists of *N_tr_* triples of (12)–(14).

**Figure 6:**
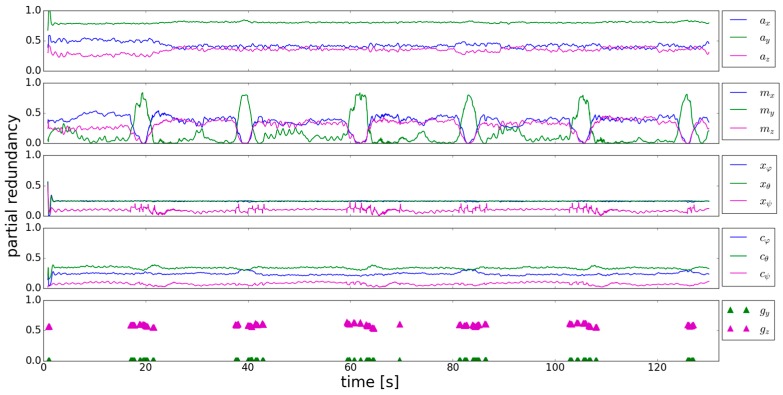
Partial redundancy of the actual trajectory when using two times the same trajectory data.

In this article additional trajectories are simulated, which means that they are retrieved from the trajectory considered in this section by performing rotations in the same manner as done in (49). This is a simplification respectively a synthetical example whose aim is to retrieve more insights into how additional observations affect the partial redundancy. Before using additional rotated trajectories, the effect of using the same trajectory data two times will be analyzed. The reason is that no additional roll and pitch angles have to be estimated and therefore the results for the partial redundancy can be directly compared to the results from Section 3.1 respectively Figure 4. The results of the partial redundancy of the observations from the actual trajectory are shown in Figure 6.

The most obvious effect can be seen in the turn detection. The gyroscope is used less in the straight trajectory parts, which leads to slightly different appearance of the *r_i_*. Nevertheless, the height of the *r_i_* can be compared. The *r_i_* of the previously estimated roll and pitch angel has not changed. It can be seen, that the *r_i_* of the accelerometer, the system noise components and the previously estimated yaw angle are raised by using additional observations, which is especially important for the system noise of the yaw angle. The controllability of this quantity is now slightly improved. The components of the magnetometer and the gyroscope, which already had quite high *r_i_* are now also raised, whereas an effect on the low *r_i_* during turns is not visible.

**Figure 7:**
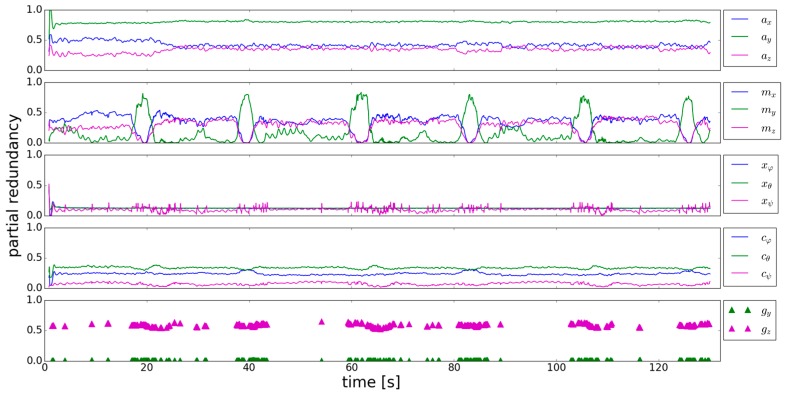
Partial redundancy of the actual trajectory when using additional data from one simulated, rotated trajectory.

This is not a surprise, as trajectory data is used two times where the controllability of these quantities is suboptimal. Figure 7 shows the partial redundancy of the observations from the actual trajectory by using one additional trajectory, where the smartphone’s orientation is different. This additional trajectory comes from rotating the trajectory data as mentioned above (49), which is a simplification respectively simulation. In a real-life application some sort of search-algorithm would have to be performed on the stored trajectories, to find the ones where users took the same path as in the actual trajectory. The only thing that has changed, are the *r_i_* of the previously estimated roll and pitch angles, as they are clearly reduced now. The reason could be that the overall redundancy has not changed from the scenario in Figure 6 to the scenario in Figure 7 but more parameters have to be estimated. The remaining *r_i_* have not changed. Especially *r_g-y_* is still close to zero, despite using an additional trajectory where the smartphone’s orientation is different. The functional relation of the four gyroscope measurements—resulting from using the actual and the simulated trajectory (i.e., the two formulas of type (4))—doesn’t lead to an improved mutual controllability. The same holds for the *r_i_* of the magnetometer measurements which are close to zero.

**Figure 8:**
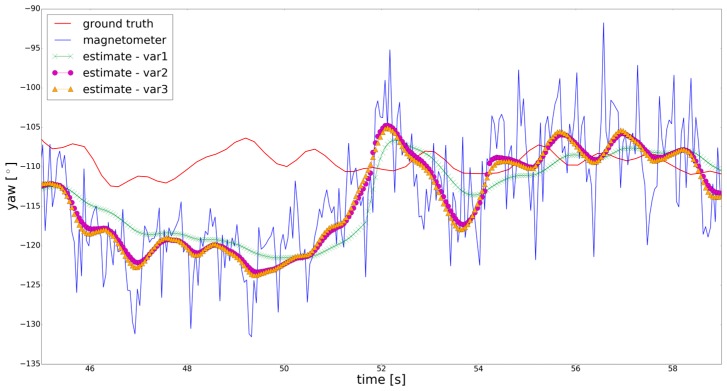
Different results for the yaw angle compared to ground truth from total station. In estimation variant 1 the original standard deviations from Table 2 are used. Variant 2 uses the adapted standard deviations. Variant 3 uses one simulated additional trajectory (equal to the one of Figure 7).

Figure 8 shows the ground truth from total station (see Section 2.2), the raw result from the magnetometer and different variants of the estimated yaw angle. For better visibility, again a representative section of the trajectory is chosen. Estimation variant 1 clearly differs from the rest of the estimation variants as it is much smoother. In this variant the original standard deviations from Table 2 are used, whereas in the other variants the adapted ones are used. The differences between variant 2 and 3 are negligible. This is reasonable, as the additional trajectory data of variant 3 is just rotated in comparison to the original trajectory data. Thus, the additional trajectory data used in this article does not contribute to the state estimation process but to the improvement of the inner reliability. In future experiments, real multiple trajectory data has to be collected from different smartphones and users to further evaluate the approach presented in this section. In this article, variant 1 performs slightly better with the drawback of worse inner reliability. In the beginning of the trajectory section shown in Figure 8, the raw result from magnetometer as well as the estimated yaw angles are shifted due to undetected systematic deviations. The aim of future research will be to detect such systematic deviations based on improved inner reliability, enabling better performance of a Kalman filter used for orientation determination.

## 4. Discussion

The main novelty of this article is the formulation of the Kalman filter and the redundancy matrix in the GHM. Depending on the system description in the system equation two different sets of update equations are derived, which are both applied to orientation determination as well as to partial redundancy calculation. The results of these derivations are used to analyze the inner reliability based on the partial redundancy. Analyzing the partial redundancy shows that in the Kalman filter used in Section 2.1 the system equation contribute disproportionately highly to the estimation of the orientation compared the measurement equation. This means that the observations of the system equation are nearly uncontrolled. Two general ways are considered to intervene into the inner reliability respectively partial redundancy.

First, effects of changes in the stochastic model are analyzed. By adapting the standard deviations, the group redundancies can be improved. Analyzing the partial redundancy of the individual observations shows that observations which crucially determine the orientation are still badly controlled. Systematic deviations in such observations can cause huge disturbances in the estimated quantities. By increasing and decreasing the standard deviations, their influence on the partial redundancy was analyzed. It appears that reducing the standard deviation of the magnetometer’s y-component, could increase many partial redundancies but its controllability is bad. The y-component of the gyroscope as well as the system noise of the yaw angle cannot be controlled by observations of the actual trajectory. It has to be mentioned that the resulting insights are in a way limited to the considered trajectory. Such adaptions in the stochastic model have to be also tested in other trajectories and different devices.

Furthermore, effects of changes in the functional model on the partial redundancy are considered. In a first step the influences of the smartphone’s orientation on the partial redundancy were analyzed. Rotating the trajectory data shows that if more than one sensor axis is sensing the quantity of interest, the controllability will be improved. Using multiple trajectory data should take advantage of this behavior. The formulation of the Kalman filter update equations in the GHM enables processing multiple trajectory data in a Kalman filter. Partial redundancy is improved through this approach but still there are critical observations, such as the y-components of the gyroscope and magnetometer. Just using more data does not mandatory lead to better controllability in the GHM. Additionally, improving inner reliability does not lead to better accuracy of the estimated quantities. These questions respectively challenges will be addressed in future studies.

## Figures and Tables

**Table 1:** Comparison of the Kalman filter update equations.

	Gauss-Markov	Gauss-Helmert (Simplification)	Gauss-Helmert
d	$l_{m}-f_{m}\left(\bar{x}\right)$	$-w_{m}=-f_{m}\left(l_{m},\bar{x}\right)$	$\left[\begin{array}{c} -w_{s}\\ -w_{m} \end{array}\right]=\left[\begin{array}{c} -f_{s}\left(l_{s},\bar{x}\right)\\ -f_{m}\left(l_{m},\bar{x}\right) \end{array}\right]$
D	$A_{m}\sum_{\bar{x}\bar{x}}A_{m}^{T}$	$B_{m}\sum_{ll,m}B_{m}^{T}+A_{m}\sum_{\bar{x}\bar{x}}A_{m}^{T}$	$\left[\begin{array}{cc} D_{s} & 0\\ 0 & D_{m} \end{array}\right]=$ $=\left[\begin{array}{cc} B_{s}\sum_{ll,s}B_{s}^{T}+A_{s}\sum_{\hat{x}\hat{x},m}A_{s}^{T} & 0\\ 0 & B_{m}\sum_{ll,m}B_{m}^{T}+A_{m}\sum_{\hat{x}\hat{x},s}A_{m}^{T} \end{array}\right]$
K	$\sum_{\bar{x}\bar{x}}A_{m}^{T}D^{-1}$		$\left[\begin{array}{cc} K_{s} & K_{m} \end{array}\right]=\left[\begin{array}{cc} \sum_{\hat{x}\hat{x},m}A_{s}^{T}D_{s}^{-1} & \sum_{\hat{x}\hat{x},s}A_{m}^{T}D_{m}^{-1} \end{array}\right]$
$\sum_{\hat{x}\hat{x}}$	$\left(E-KA_{m}\right)\sum_{\bar{x}\bar{x}}$		$\left(E-K_{m}A_{m}\right)\sum_{\hat{x}\hat{x},s}=\left(E-K_{s}A_{s}\right)\sum_{\hat{x}\hat{x},m}$
$\hat{x}$	$\bar{x}+Kd$		

**Table 2:** Standard deviations used for the sensor measurements and system noise.

Standard Deviations	Gyroscope	System Noise	Accelerometer	Magnetometer
Original	30°/s	10°/s	1 m/s^2^	5 μT
Adapted	60°/s	20°/s	0.5 m/s^2^	2.5 μT

**Table 3:** Influence of the change of the standard deviation of one observation on the partial redundancy of the other observations. “+” indicates a raise and “−” a reduction of the partial redundancy. At the left sign, the standard deviation was increased by the factor 10 and at the right sign it was decreased by the factor 0.1. Grey shaded cells show the change in the corresponding observation and green means that the observations are functionally related.

	*σ_g-y_*	*σ_g-z_*	*σ_c-ϕ_*	*σ_c-θ_*	*σ_c-ψ_*	*σ_a-x_*	*σ_a-y_*	*σ_a-z_*	*σ_m-x_*	*σ_m-y_*	*σ_m-z_*
*r_x-ϕ_*			−|−				−|+				
*r_x-θ_*				−|−		−|+		−|+			
*r_x-ψ_*		+|−			+|				−|+	−|+	−|+
*r_g-y_*	+|										
*r_g-z_*		+|−							−|+	−|+	−|+
*r_c-ϕ_*			+|−				−|+			|+	
*r_c-θ_*				+|−		−|+		−|+		|+	
*r_c-ψ_*					+|−						
*r_a-x_*				−|+		+|−		−|+			
*r_a-y_*			−|+				+|−				
*r_a-z_*				−|+		−|+		+|−		|+	
*r_m-x_*		−|+							+|−	−|+	−|+
*r_m-y_*		−|+							−|+	+|−	−|+
*r_m-z_*		−|+							−|+	−|+	+|−

## Appendix A

Formula (31) will be derived by rearranging (30):

(A1) $$0=f({\hat{x}}_{k-1},u_{k},c_{k})-{\bar{x}}_{k}$$

This is the approximate solution of the following least squares parameter estimation problem:

(A2) $$0=f({\hat{\hat{x}}}_{k-1},{\hat{u}}_{k},{\hat{c}}_{k})-{\hat{x}}_{k}$$

This is a special case of the GHM (22). Using the approximate solution for Taylor linearization—and neglecting second and higher order terms—yields:

(A3) $$0=f({\hat{x}}_{k-1},u_{k},c_{k})-{\bar{x}}_{k}-E\left({\hat{x}}_{k}-{\bar{x}}_{k}\right)+\frac{\partial f}{\partial {\hat{x}}_{k-1}}\left({\hat{\hat{x}}}_{k-1}-{\hat{x}}_{k-1}\right)+\frac{\partial f}{\partial u_{k}}\left({\hat{u}}_{k}-u_{k}\right)+\frac{\partial f}{\partial c_{k}}\left({\hat{c}}_{k}-c_{k}\right)$$

Taking (30) into account, the first two terms are equal to zero. The next step is to insert the following quantities in the linearized functional model above:

(A4) $$\begin{array}{l} \frac{\partial f}{\partial {\hat{x}}_{k-1}}=T_{k,k-1}\\ \frac{\partial f}{\partial u_{k}}=U_{k,k-1}\\ \frac{\partial f}{\partial c_{k}}=C_{k,k-1}\\ {\hat{\hat{x}}}_{k-1}-{\hat{x}}_{k-1}=v_{{\hat{x}}_{k-1},k}\\ {\hat{u}}_{k}-u_{k}=v_{u,k}\\ {\hat{c}}_{k}-c_{k}=v_{c,k}\\ \Delta {\hat{x}}_{k}={\hat{x}}_{k}-{\bar{x}}_{k} \end{array}$$

Insertion gives:

(A5) $$0=-E\Delta {\hat{x}}_{k}+T_{k,k-1}v_{{\hat{x}}_{k-1},k}+U_{k,k-1}v_{u,k}+C_{k,k-1}v_{c,k}$$

The last step is to summarize ***T**_k,k_*_−1_, ***U**_k,k_*_−1_ and ***C**_k,k_*_−1_ in one matrix and $v_{\hat{x},k-1,k}$, $v_{u,k}$ and $v_{c,k}$ in one vector:

(A6) $$-E\Delta {\hat{x}}_{k}+\left[\begin{array}{ccc} T_{k,k-1} & U_{k,k-1} & C_{k,k-1} \end{array}\right]\left[\begin{array}{c} v_{{\hat{x}}_{k-1},k}\\ v_{u,k}\\ v_{c,k} \end{array}\right]=0$$

## Appendix B

The following calculation rules will be used to calculate the overall redundancy [42]:

(A7) $$\begin{array}{l} tr\left(AB\right)=tr\left(BA\right)\\ tr\left(A+B\right)=tr\left(A\right)+tr\left(B\right) \end{array}$$

Calculation of the trace of ***R***:

(A8) $$\begin{array}{l} tr\left(R\right)=tr\left(\sum_{ll}B^{T}{\left(B\sum_{ll}B^{T}\right)}^{-1}\left(E-A\sum_{\hat{x}\hat{x}}A^{T}{\left(B\sum_{ll}B^{T}\right)}^{-1}\right)B\right)=\\ tr\left({\left(B\sum_{ll}B^{T}\right)}^{-1}\left(E-A\sum_{\hat{x}\hat{x}}A^{T}{\left(B\sum_{ll}B^{T}\right)}^{-1}\right)B\sum_{ll}B^{T}\right)=\\ tr\left({\left(B\sum_{ll}B^{T}\right)}^{-1}\left(B\sum_{ll}B^{T}-A\sum_{\hat{x}\hat{x}}A^{T}\right)\right)=tr\left(E\right)-tr\left({\left(B\sum_{ll}B^{T}\right)}^{-1}A\sum_{\hat{x}\hat{x}}A^{T}\right) \end{array}$$

The identity matrix comes from the matrix product ***B**Σ_ll_**B**^T^* and its inverse. ***B*** contains the derivatives of the condition equations with respect to the observations. Therefore, its dimension is *b* × *n*, where *n* equals the number of observations and *b* equals the number of condition equations. Hence, the identity matrix has dimension *b* × *b* and its trace corresponds to the number of condition equations:

(A9) $$\begin{array}{l} tr\left(R\right)=b-tr\left({\left(B\sum_{ll}B^{T}\right)}^{-1}A\sum_{\hat{x}\hat{x}}A^{T}\right)=b-tr\left(A^{T}{\left(B\sum_{ll}B^{T}\right)}^{-1}A\sum_{\hat{x}\hat{x}}\right)=\\ b-tr\left(A^{T}{\left(B\sum_{ll}B^{T}\right)}^{-1}A{\left(A^{T}{\left(B\sum_{ll}B^{T}\right)}^{-1}A\right)}^{-1}\right)=b-tr\left(E\right) \end{array}$$

As ***A*** contains the derivatives of the condition equations with respect to the parameters, the identity matrix has dimension *u* × *u*, where *u* equals the number of parameters. Thus, the trace of ***R*** equals the number condition equations minus the number of parameters, which is the overall redundancy in the GHM [34].

(A10) tr(R)=b−u=r

## Appendix C

To derive (47) and (48), the following quantities are used to evaluate (18):

(A11) $$\begin{array}{l} \sum_{uu,k}=\left[\begin{array}{ccc} \sigma_{g-x}^{2} & 0 & 0\\ 0 & \sigma_{g-y}^{2} & 0\\ 0 & 0 & \sigma_{g-z}^{2} \end{array}\right]=\sigma_{g}^{2}E\\ U_{k,k-1}=\left[\begin{array}{ccc} 0 & 0 & 0\\ 0 & 0 & 0\\ 0 & \sin\varphi \frac{\Delta t}{\cos\theta } & \cos\varphi \frac{\Delta t}{\cos\theta } \end{array}\right]\\ A_{m,k}=-E\\ D_{k}^{-1}=\left[\begin{array}{ccc} d_{11}^{*} & d_{12}^{*} & d_{13}^{*}\\ d_{21}^{*} & d_{22}^{*} & d_{23}^{*}\\ d_{31}^{*} & d_{32}^{*} & d_{33}^{*} \end{array}\right] \end{array}$$

***U**_k,k_*_−1_ contains the derivatives of (1), (2) and (4) with respect to the gyroscope measurements. ***A**_m,k_* contains the derivatives of (12)–(14) with respect to the parameters respectively the Euler angles and therefore equals the negative identity matrix The variances of the three gyroscope axes are assumed to be equal. Inserting these quantities into (12) gives:

(A12) $$\sum_{j=1}^{n_{u}}r_{u,k,j}=\sigma_{g}^{2}\cdot \sum_{j=1}^{n_{u}}e_{j}^{T}E\left[\begin{array}{ccc} 0 & 0 & 0\\ 0 & 0 & \sin\varphi \frac{\Delta t}{\cos\theta }\\ 0 & 0 & \cos\varphi \frac{\Delta t}{\cos\theta } \end{array}\right](-E)\left[\begin{array}{ccc} d_{11}^{*} & d_{12}^{*} & d_{13}^{*}\\ d_{12}^{*} & d_{22}^{*} & d_{23}^{*}\\ d_{13}^{*} & d_{23}^{*} & d_{33}^{*} \end{array}\right](-E)\left[\begin{array}{ccc} 0 & 0 & 0\\ 0 & 0 & 0\\ 0 & \sin\varphi \frac{\Delta t}{\cos\theta } & \cos\varphi \frac{\Delta t}{\cos\theta } \end{array}\right]e_{j}$$

Evaluating the expression between the identity vectors ***e**_j_* gives:

(A13) $$\sum_{j=1}^{n_{u}}r_{u,k,j}=\frac{\sigma_{g}^{2}\cdot d_{33}^{*}\cdot \Delta t^{2}}{\cos^{2}\theta }\cdot \sum_{j=1}^{n_{u}}e_{j}^{T}\left[\begin{array}{ccc} 0 & 0 & 0\\ 0 & \sin^{2}\varphi & \sin\varphi \cdot \cos\varphi \\ 0 & \sin\varphi \cdot \cos\varphi & \cos^{2}\varphi \end{array}\right]e_{j}$$
